# Elevated level of extracellular vimentin is associated with an increased fibrin formation potential in sepsis: ex vivo swine study

**DOI:** 10.1186/s40635-024-00660-5

**Published:** 2024-08-29

**Authors:** Marina Martinez-Vargas, Arun Saini, Subhashree Pradhan, Luis Gardea, Barbara Stoll, Inka C. Didelija, K. Vinod Vijayan, Trung C. Nguyen, Miguel A. Cruz

**Affiliations:** 1 Department of Medicine, Baylor College of Medicine, Houston, TX USA; 2 Center for Translational Research On Inflammatory Diseases at the Michael E. DeBakey Veterans Affairs Medical Center, Houston, TX USA; 3 Division of Critical Care Medicine, Department of Pediatrics, Baylor College of Medicine/Texas Children’s Hospital, Houston, TX USA; 4 US Department of Agriculture/Agricultural Research Service, Children’s Nutrition Research Center, Houston, TX USA; 5 Section of Cardiovascular Research, Baylor College of Medicine and MEDVAMC, 2002 Holcombe, Bldg. 109, R-146, Houston, TX 77030 USA

**Keywords:** Large animal model, Sepsis, Coagulopathy, Fibrin, Vimentin

## Abstract

**Background:**

Sepsis can lead to coagulopathy and microvascular thrombosis. Prior studies, including ours, reported an increased level of extracellular vimentin in blood derived from septic patients. Moreover, we identified the contribution of extracellular vimentin to fibrin formation and to the fibrin clot structure ex vivo in plasma from septic patients. Here, we tested the status of plasma vimentin and its impact on fibrin clots using our recently described swine model of methicillin-resistant Staphylococcus aureus (MRSA) sepsis-induced coagulopathy.

**Results:**

We employed ELISA, size-exclusion chromatography, vimentin antibodies, confocal microscopy, and turbidity assays on piglet plasma obtained at pre- and post-MRSA inoculation. Plasma vimentin level at 70 h post-MRSA inoculation was on average twofold higher compared to pre-infection (0 h) level in the same animal. Anti-vimentin antibody effectively reduced fibrin formation ex vivo and increased porosity in the fibrin clot structure generated from septic piglet plasma. In contrast to plasma at 0 h, the size-exclusion chromatography revealed that phosphorylated vimentin was in-complex with fibrinogen in septic piglet plasma.

**Conclusions:**

Thus, our swine model of sepsis-induced coagulopathy, reproduced increased extracellular circulating vimentin and subsequent potentiation of fibrin formation, often observed in septic patient. These outcomes validate the use of large animal models to investigate the dysregulated host immune response to infection leading to coagulopathy, and to develop new therapies for sepsis-induced disseminated microvascular thrombosis.

**Supplementary Information:**

The online version contains supplementary material available at 10.1186/s40635-024-00660-5.

## Background

Sepsis-induced coagulopathy may lead to disseminated intravascular coagulation (DIC) that is associated with high mortality and currently, only supportive therapies are available [1, 2]. One possible explanation for the lag in the development of effective therapeutic strategies is the need for clinically relevant large animal models for the study of sepsis [3, 4]. Recently, we reported a hemodynamically and clinically relevant pediatric swine model with human MRSA sepsis-induced coagulopathy that can last for 70 h. Among other host responses to the insult, the model can be used to study coagulopathy to infection and identify potential early biomarkers [5].

An observational study reported that high levels of serum vimentin in pediatric patients with severe sepsis predicts a high-risk hospital mortality in these patients [6]. Another clinical study supports the use of serum vimentin level as a biomarker for diagnosing and predicting the prognosis of sepsis [7]. The same study reports that vimentin has a role in lymphocyte apoptosis and in the inflammatory response. Together, these reports suggest that extracellular vimentin serves as a potential biomarker in sepsis and may be considered for early identification and timely therapeutic interventions for patients with severe sepsis. Previously, we also described the elevated levels of extracellular (plasma) vimentin in the circulation of patients with sepsis [8]. Moreover, we characterized and described the novel function of vimentin in fibrin polymerization ex vivo using plasma from critically ill patients with immune dysregulation and sustained systemic inflammation [8]. Although low levels of plasma vimentin were observed in healthy subjects, the vimentin detected in septic patients appears to be distinct from that found in healthy subjects [8]. Thus, our data suggested that elevated levels of an aberrant vimentin contribute to coagulopathy by directly interacting with fibrinogen and enhancing fibrin formation in patients with sepsis. However, it remains undefined if the expression of this abnormal vimentin is directly linked to the dysregulated host immune response and sustained systemic inflammation induced by sepsis. Therefore, this study aimed to determine if our swine model of sepsis-induced coagulopathy replicates the increment of a distinct plasma vimentin and to assess the capacity of anti-vimentin on reducing fibrin formation ex vivo in the swine plasma.

## Methods

### Plasma from the swine sepsis studies

The plasma samples used in this study were obtained from piglets in a previous study that established sepsis-induced coagulopathy following MRSA infection [5]. Briefly, we used blood samples collected from arterial catheters in 2 male and 2 female domestic piglets (4 wk old, 8 kg body weight, approximately early toddler age equivalent in human) at pre- and 70 h post-sepsis time points. Four days prior to the sepsis experiment, the piglets received anesthesia (isoflurane: 5% induction and 1–2% maintenance by face mask) during the surgical implantation of the telemetry device. Buprenorphine sustained release (SR) was given subcutaneously to manage postoperative pain. After surgery, the piglets recovered in their cages for 4 days, and were monitored with telemetry device to acquired baseline vital signs. Prior to MRSA inoculation, the piglets received buprenorphine SR (0.12–0.24 mg/kg) subcutaneously to minimize pain, discomfort and distress. During the sepsis experiment, the piglets received Tylenol intravenously every 6 h as needed for persistent fever (> 41 degree Celsius for > 1 h). Because this was an awake MRSA sepsis model, the piglets did not receive anesthesia during the sepsis experiment. Piglets were euthanized immediately if they became moribund (lying laterally recumbent and unresponsive to stimuli), showed severe acute respiratory distress, or suffered from inability to tolerate appropriate intake due to abdominal distention, emesis, or decreased consciousness or physical injury. No early euthanasia was necessary in our study, and piglets were euthanized according to the scientific protocol (70-h post-inoculation). All animal procedures were reviewed and approved by the Baylor College of Medicine Animal Care and Use Committee (study number: AN-6148 Arginine Metabolism in Sepsis; approved May 1, 2014, to August 24, 2022) [5].

### Reagents

Human fibrinogen was obtained from Calbiochem, and thrombin from Sigma (St. Louis, MO). The rabbit anti-vimentin antibody was purchased from Proteintech (Rosemont, IL). This rabbit anti-vimentin antibody was validated as previously reported [8]. Isotype immunoglobulin (IgG) was purchased from Sigma. To detect vimentin phosphorylation, we use a rat monoclonal anti-Ser71 phospho-vimentin antibody (clone KAM-cc250) from Enzo Life Science.

### Gel filtration chromatography

The pre- and post-MRSA plasma samples were subjected to size-exclusion chromatography using a Superose 6^™^ column as described [8]. The collected fibrinogen fractions were analyzed for the presence of associated aberrant vimentin by immunoblotting with antibodies to phospho Ser71 and total vimentin.

### Fibrin polymerization assays

Fibrin formation and degradation were performed as we described [8, 9]. Briefly, 5% plasma in Tris-buffered saline (TBS) (50 mmol/l Tris, 0.15 mol/l NaCl, pH 7.4) was mixed with anti-vimentin antibody or isotype IgG in the presence or absence of tissue plasminogen activator (tPA, 250 ng/ml, Cathflo Activase) and recombinant pig tissue plasminogen activator (pig tPA, 5 ng/ml, Abcam, ab92728). Polymerization and/or fibrinolysis were initiated with the addition of 0.25 U of human thrombin. The enzymatic reaction was evaluated by tracking turbidity using a spectrophotometer set to 350 nm.

### Imaging the fibrin clot structure

To aid in imaging plasma was supplemented with 2% (w/w) of human fibrinogen conjugated to Alexa Fluor 647 (Thermo Scientific). Clot formation was initiated with the addition of 1U thrombin (EMD Millipore) in the presence of 2.4 mM calcium as described [8, 9]. For vimentin detection in the fibrin structure, we followed a protocol described [8].

### Fibrin porosity

As described [8, 10], the fibrin clot was visualized with confocal microscopy*,* and porosity was quantified using Image J software.

### ELISA to measure plasma vimentin

Vimentin levels were determined using an ELISA kit for pig vimentin in plasma (LS Bio, # LS-F15720), following the instructions of the supplier.

### Statistical analysis

GraphPad Prism 8 software (San Diego, CA) was used to perform statistical analyses. Comparisons between groups were conducted by paired t-test. P values were 2-sided, and statistical significance was determined by a P value < 0.05.

## Results

**Fig. 1:**
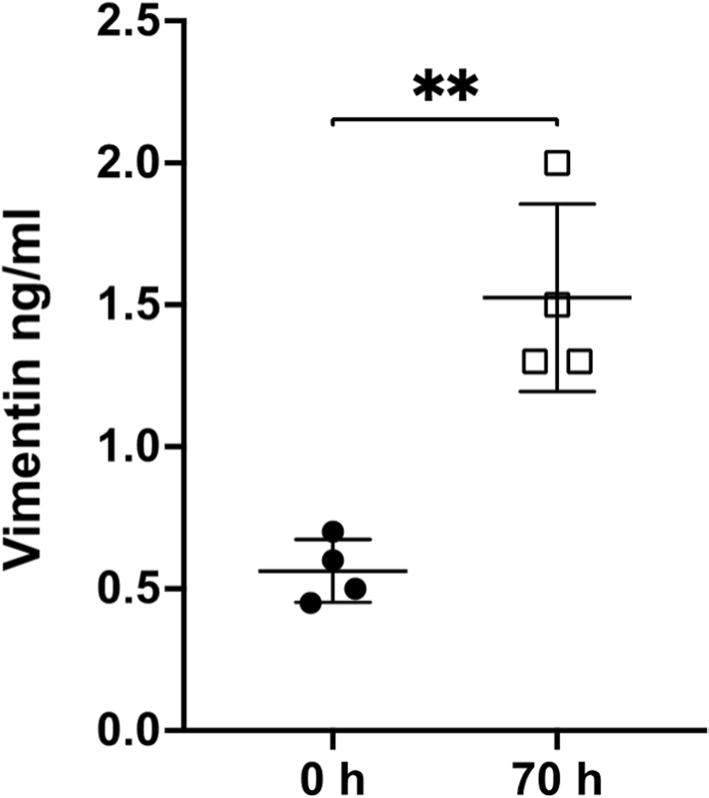
Elevated levels of plasma vimentin upon MRSA infection in swine model of coagulopathy. Levels of plasma vimentin in pre-infected swine were 0.56 ± 0.11 ng/ml and 1.5 ± 0.33 ng/ml for post-MRSA. Paired two-tailed t-tests were performed, mean ± SD, **p < 0.0032. *n* = 4 animals were analyzed in duplicates

*Infection with MRSA increased extracellular vimentin in the swine model of sepsis-induced coagulopathy.* Here, we used the swine model of MRSA sepsis-induced coagulopathy to study the interplay between plasma vimentin and fibrinogen. We previously reported increased fibrinogen level in post-MRSA infected piglet (70 h, ~ 400 mg/dL versus 0 h, ~ 100 mg/dL) [5]. This model also offers the advantage of performing comparative analysis of plasma vimentin from the same animal, at pre- and post-infection. By using ELISA, we observed that the septic animals had significantly elevated plasma vimentin levels, compared to that of pre-infected piglets as shown in Fig. 1 (post-infected 1.5 ± 0.33 ng/ml *vs*. pre-infected piglets 0.56 ± 0.11 ng/ml (*n* = 4), mean ± SD, **p < 0.0032).

**Fig. 2:**
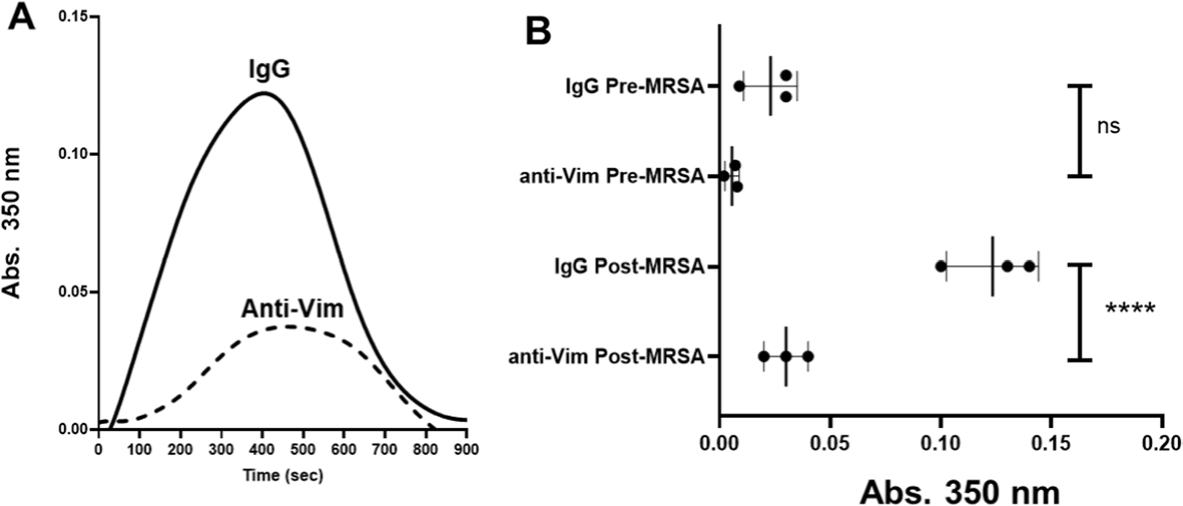
Anti-vimentin (Vim) antibody affected ex vivo fibrin formation in swine plasma. **A** The representative curve for fibrin formation and fibrinolysis using porcine plasma from post-MRSA inoculation. Fibrin formation and fibrinolysis were initiated with thrombin, calcium, and in the presence of recombinant tissue-type plasminogen activator, anti-vimentin (Vim) antibody or isotype IgG (2.5 g/ml). Turbidity was measured at 350 nm. **B** The anti-Vim antibody had a modest (not significant) effect in plasma at pre-MRSA as compared to plasma with isotype IgG. In contrast, in plasma from post-MRSA inoculation, the anti-Vim antibody significantly reduced the maximal absorbance (peak of fibrin formation), to 3.0 × 10^–2^ ± 0.01 mean ± SD, *n* = 3 compared with isotype IgG, 12.3 × 10^–2^ ± 0.015 mean ± SD (*n* = 3, two-tailed ****p < 0.0001)

*Anti-vimentin antibody reduced the fibrin formation potential and increased fibrin clot porosity in plasma from the septic piglets.* We have previously shown that extracellular vimentin can engage fibrinogen and modulate fibrin polymerization in patients with sepsis [8]. Therefore, we tested whether the elevated vimentin observed in septic piglets was capable of markedly influencing fibrin structures in post-MRSA plasma samples compared to pre-infection. We used anti-vimentin antibody to block the effect of extracellular vimentin on fibrin polymerization and degradation ex vivo. The anti-vimentin antibody significantly reduced fibrin formation in post-infection plasma with a maximum absorbance (optical density) of 3.0 × 10^–2^ ± 0.015, mean ± SD (*n* = 3) compared with IgG isotype antibody with a maximum absorbance of 12.3 × 10^–2^ ± 0.015 mean ± SD (*n* = 3) (Fig. 2A and B). As expected, the anti-vimentin antibody had a greater inhibitory effect on plasma from the septic piglets, compared to plasma from pre-infected piglets, indicating a diminished contribution of vimentin to clot formation before MRSA infection. Together, these results indicate that the elevated vimentin observed in septic piglet plasma can support enhanced fibrin polymerization as we previously observed with septic patients [8].

**Fig. 3:**
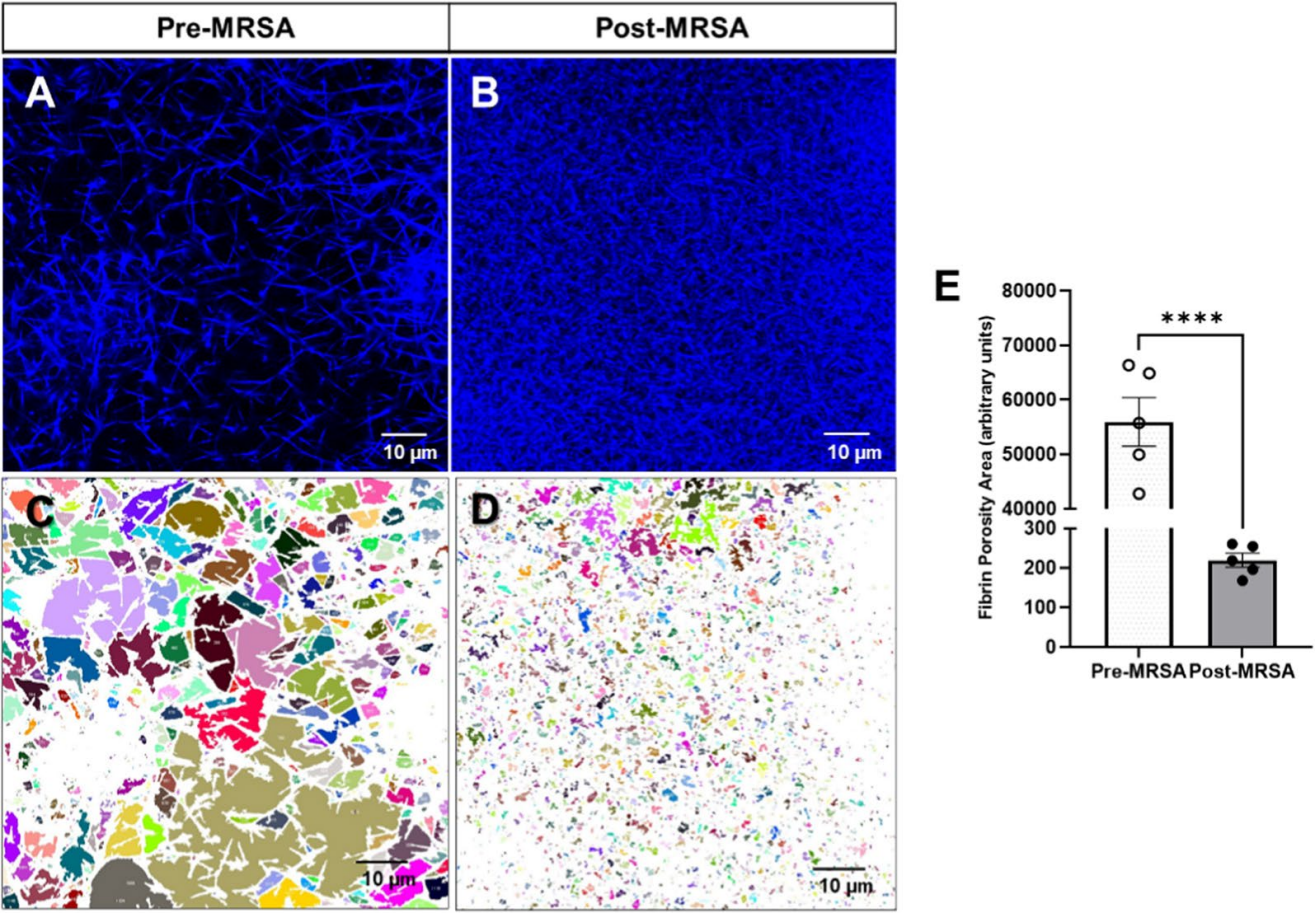
MRSA swine infection reduced porosity of fibrin clot structure in plasma. Representative confocal microscopy images of fibrin clots of pig plasma spiked with fluorescent human fibrinogen (blue) at magnification of 120 × formed in plasma from **A** pre- or **B** post-infected piglet. Confocal microscopy image shows an increment in the fibrin mesh and a decrease in the porosity area (sizes of unoccupied areas by fibrin shown with variety of colors) in plasma of the post-infected piglet (**B**, **D**). Porosity represented by the colors (**C**, **D**) and **E** shows quantification of porosity. Paired t-test analysis of fibrin porosity area (n = 5, two-tailed ****p < 0.0001)

Previously, we also reported that the fibrin fibers diameter and porosity were compromised in critically ill septic patients [5]. Therefore, we next performed comparative analysis of clot structures between plasma obtained before and after the MRSA infection of the same animal as shown in Fig. 3A and B. The clot formed with pre-infected plasma resulted in larger pores (colored area unoccupied by fibrin) compared to the clot formed with post-MRSA infected plasma **(**Fig. 3C–E**).** These results demonstrated that during sepsis, the fibrin clot became denser with a decrease in porosity, thus validating our previous observations using plasma from septic patients [8].

**Fig. 4:**
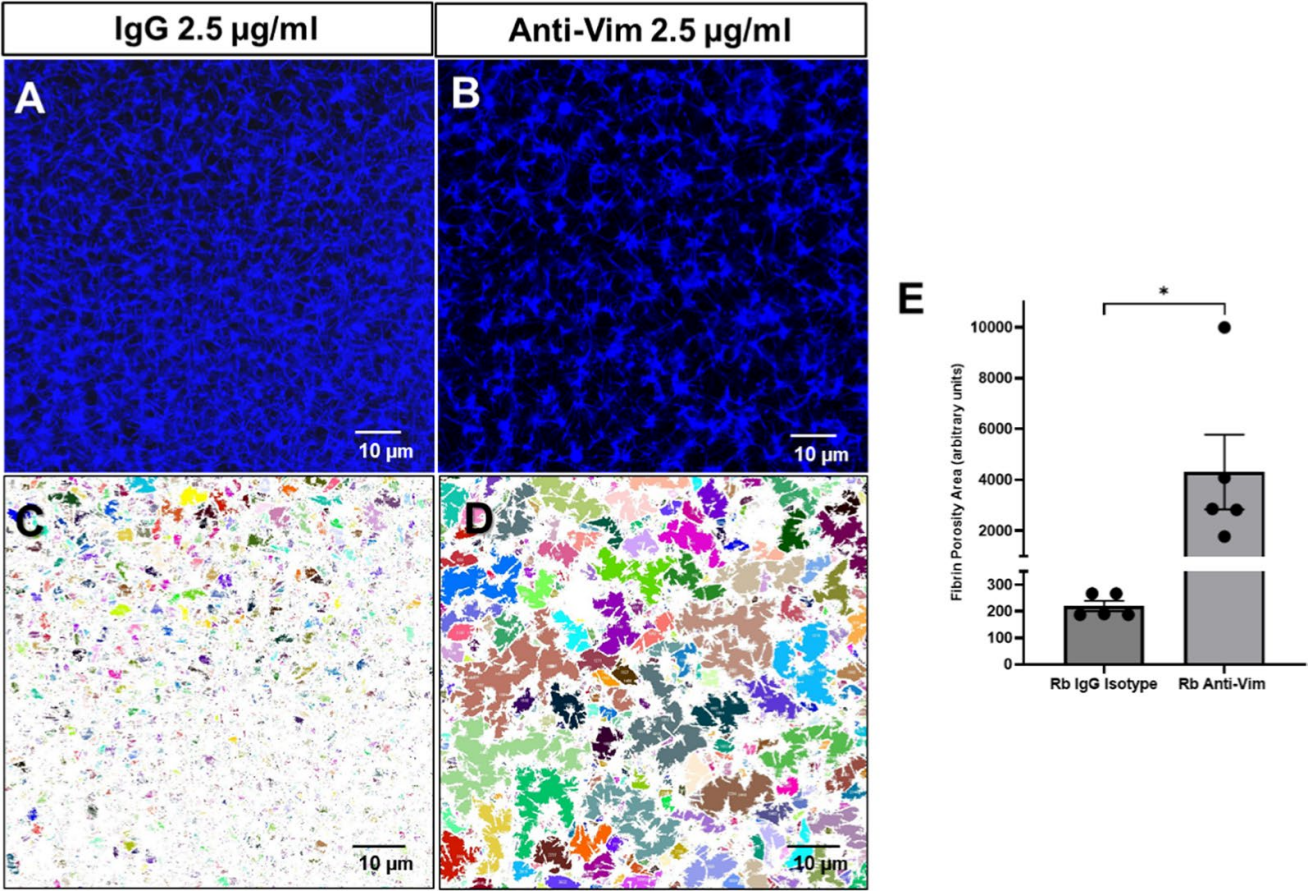
Anti-Vim antibody induced changes in the fibrin clot structure in plasma of septic piglets. **A**, **B** Representative confocal microscopy images of clot structure in pig plasma, spiked with fluorescent human fibrinogen. **B** Shows a clot with a reduced fibrin mesh and increased porosity area in plasma of the septic piglet (**D**). **C** and **D** were used to analyze porosity as in Fig. 3. **E** Addition of anti-Vim antibody significantly increased porosity area in post-MRSA. Shown paired t-test analysis of fibrin porosity area from **C** and **D** with SEM. Two-tailed *p < 0.04 *n* = 5

Next, we investigated the effect of blocking plasma vimentin on the resultant fibrin network structure. Figure 4A and B shows representative clot structures generated with post-infected piglet plasma in the presence of isotype IgG (2.5 μg/ml) and anti-vimentin (2.5 μg/ml) antibodies, respectively. We quantified the sizes of the fibrin porosity area as shown in Fig. 4C–E. The analysis showed that the anti-vimentin antibody increased the porosity area in the resultant fibrin clot structure compared with isotype IgG. The data from septic piglets confirmed our previous report in which anti-vimentin antibody incremented fibrin porosity ex vivo in plasma from septic patients [8].

**Fig. 5:**
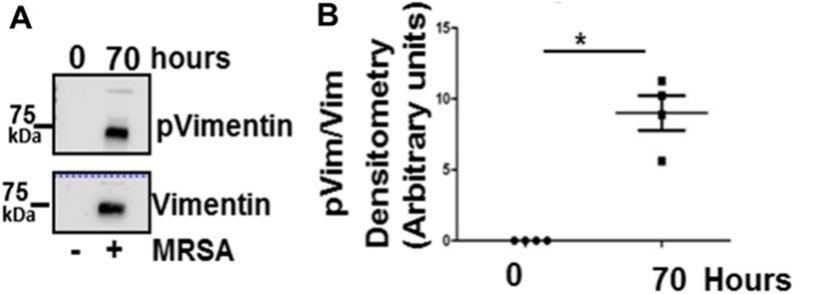
Phosphorylated form of plasma vimentin (vim) associated with fibrinogen in septic plasma. Plasma from piglets at 0 h or 70 h after MRSA inoculation was subjected to size-exclusion chromatography as previously described in reference 7. **A** Immunoblot of Ser 71 phosphorylated (p) vimentin (upper panel). Blot was stripped and reprobed with anti-vimentin antibody (lower panel). **B** Densitometry analysis as a ratio of phosphorylated p71 vimentin (pVim)/Vim is shown. Paired t-test *p < 0.05

*An aberrant plasma vimentin was identified to associate with fibrinogen in the swine model of sepsis-induced coagulopathy.* As we described earlier [8] pre- and 70 h post-infected plasma samples were fractionated by size-exclusion chromatography and the fibrinogen eluate analyzed for the presence of associated vimentin. In contrast to pre-MRSA infected plasma sample, vimentin co-eluted with fibrinogen from post-MRSA infected plasma (Fig. 5A). Since phosphorylation of vimentin destabilizes the filamentous form and allows for reorganization of vimentin [11, 12], we assessed the phosphorylation status of fibrinogen-associated vimentin in septic piglets. Vimentin that co-eluted with fibrinogen from post-infected plasma was phosphorylated on Serine71 (Ser 71) as shown in Fig. 5A–B. This result suggests that in sepsis, an elevated level of a phosphorylated extracellular vimentin is in-complex with fibrinogen.

## Discussion

These findings from experimental large animal model strongly support our initial report on the new interplay between extracellular vimentin and fibrinogen in critically ill patients with dysregulated host immune response and systemic inflammation due to sepsis [8, 9]. Studies with septic patients face limitations due to the varying stages of the disease that occur before the arrival of the patients to the hospital. Our swine model offers the advantage of monitoring the progression of the disease from before the bacterial insult until the peak of illness or 70 h in our model. To test if the large animal model replicates a human manifestation of the dysregulated host immune response leading to coagulopathy due to sepsis, we investigated the level of plasma vimentin and its ability to modulate fibrin polymerization potential.

This 70 h swine model of MRSA sepsis provides a clinically relevant platform to study the evolution of the dysregulated host immune response leading to coagulopathy, frequently seen in the clinical environment [5]. In this study, we chose to analyze the levels of extracellular vimentin in plasma obtained at two-time points from the same animal (0 h and 70 h), representing pre- and post-MRSA infection. We chose the 70 h post-septic plasma samples because this was when the piglets were the sickest, which was like the analyzed septic human plasma samples from our prior study [8]. At 70 h, the piglets were the most coagulopathic (lowest platelet counts, longest aPTT, highest D-dimers, lowest activities of FV, ATIII, Protein C and ADAMTS-13) and had the lowest neurological and respiratory scores (altered mental status and tachypneic) [5]. The human plasma samples were from critically ill septic patients with a median SOFA score of 7.5, and with severe coagulopathy (thrombocytopenia, prolonged aPTT, high D-dimers, abnormal thromboelastography) [8, 9].

For this manuscript, we retrospectively adapted and calculated a published pig-specific Sequential Organ Function Assessment (pSOFA) scoring system [13]. In our swine model, we did not measure the urine output [5]. The adapted pSOFA scores at 70 h was 1–2 in our piglets (Supplemental Table 1). Our swine MRSA sepsis-induced coagulopathy, disseminated microvascular thrombosis, and early organ injuries model is an awake, non-lethal, and without hypotension sepsis model [5]. The consumptive coagulopathy and histological evidence of focal organ injuries observed in this model were not due to hypotension as the continuous mean arterial blood pressure (MAP) measurements were never below 30% from baseline (MAP > 75 mmHg throughout). We speculated that the observed coagulopathy and histological organ injuries were likely due to the dysregulated host immune response to MRSA infection. This was an early organ injuries model as it revealed that current bedside laboratory evidence of organ dysfunction, such as AST, ALT, and creatinine levels was a late sign of cellular and organ injuries. Thus, the increased extracellular circulating vimentin might give an insight into the early development of MODS.

In addition to confirming that extracellular vimentin was increased after the MRSA infection, the animal model also revealed that fibrinogen bound vimentin was robustly phosphorylated on Ser 71 (Fig. 5**).** Ser71 phosphorylation on vimentin is mediated by Rho kinase [14] and interestingly, MRSA infection induces cytoskeletal changes and activation of Rho kinase [15]. As Ser71 phosphorylation converts filamentous vimentin into soluble non-filamentous vimentin [14], it is possible that this aberrant form of vimentin may be easily secreted from cells into circulating plasma in septic piglets. Future studies will be necessary to explore whether phosphorylated Ser 71 or other post-translational modifications (PTMs) in vimentin [16] are a major determinant in the ability of aberrant vimentin to engage fibrinogen, cause changes in the fibrin formation potential and the clot structure in sepsis.

This study also confirmed that changes in the fibrin clot structure are associated with illness, including sepsis [9, 17–19]. As expected, plasma from MRSA-infected piglets validated the fact that the fibrin clot structure had a significant decrease in fibrin porosity. Notably, the addition of anti-vimentin antibody effectively increased the fibrin porosity in plasma of the sick animal. In other words, the anti-vimentin antibody could change the clot structure of septic plasma to look like that of the clot structure from healthy plasma (Fig. 4B vs. Figure 3A). Changes in fibrin porosity can affect various physiological processes such as blood flow, clot stability, and interactions with cells and proteins [17, 20]. Additionally, in medical contexts, high or low porosity of fibrin clots may be associated with certain conditions or diseases, and it can have implications for patient diagnosis, treatment, and prognosis [21–23]. Thus, one can argue that targeting fibrin clot structure with anti-vimentin antibody could be a potential therapy for patients with sepsis-induced coagulopathy in the future.

A potential drawback in this study is the use of fluorescently labeled human fibrinogen in our clot structures analyses. It is important to note that there is approximately a 70% similarity between pig and human fibrinogen (https://www.uniprot.org/blast), potentially affecting the accuracy of our findings. In fact, other studies demonstrated significant variations in the processes of clot formation and expansion, clot breakdown, and clot strength between human and pigs [24, 25]. To overcome this limitation, it will be necessary to use pig fibrinogen in the future. Another limitation of this study is the small number of animals used for our experiments. Nonetheless, we were able to detect elevated levels of aberrant vimentin in septic plasma, and a marked effect of anti-vimentin antibody on fibrin formation potential, clot structure in septic plasma, the latter being consistent with our study with critically ill septic patients [8].

Finally, this study supports the notion that elevated levels of a modified or phosphorylated extracellular vimentin during sepsis impacts fibrin formation potential and significantly modify the structure of fibrin clots. Atypical PTMs have been associated with various developmental disorders and human diseases, underscoring the significance of comprehending vimentin PTMs [16, 26]. This understanding could be valuable for stabilizing patients in clinical settings or enhancing disease detection. Further investigation is needed to understand the specific mechanisms by which an altered or different vimentin modulates thrombosis in systemic inflammation-related coagulopathy. Thus, the outcomes reported here validate the use of our large animal sepsis model to investigate the dysregulated host immune response to infection leading to coagulopathy, identify potential early biomarkers, and develop new therapies.

### Supplementary Information

Supplementary Material 1.

## Data Availability

The datasets used and/or analyzed during the current study are available from the corresponding author on reasonable request.

## Abbreviations

MRSA: Methicillin-resistant Staphylococcus aureus
DIC: Disseminated intravascular coagulation
ELISA: Enzyme-linked immunosorbent assay
tPA: Tissue plasminogen activator
PTMs: Post-translational modifications
