# Supplementary material for: Elevated level of extracellular vimentin is associated with an increased fibrin formation potential in sepsis: ex vivo swine study
Source: Intensive Care Med Exp. 2024 Aug 29;12:75. doi: 10.1186/s40635-024-00660-5 (PMC11362409; doi:10.1186/s40635-024-00660-5)
Supplement: Supplementary file 1 — Supplementary Material 1. [file 40635_2024_660_MOESM1_ESM.docx]

Supplemental Material

**Supplemental Table 1**
